# ReporType: A Flexible Bioinformatics Tool for Targeted Loci Screening and Typing of Infectious Agents

**DOI:** 10.3390/ijms25063172

**Published:** 2024-03-09

**Authors:** Helena Cruz, Miguel Pinheiro, Vítor Borges

**Affiliations:** 1 Institute of Biomedicine-iBiMED, Department of Medical Sciences, University of Aveiro, 3810-193 Aveiro, Portugal; helenaiscruz@ua.pt (H.C.); monsanto@ua.pt (M.P.); 2 Genomics and Bioinformatics Unit, Department of Infectious Diseases, National Institute of Health Doutor Ricardo Jorge (INSA), 1649-016 Lisbon, Portugal

**Keywords:** bioinformatics, sequencing, pathogen, loci screening, genotyping

## Abstract

In response to the pressing need for continuous monitoring of emergence and circulation of pathogens through genomics, it is imperative to keep developing bioinformatics tools that can help in their rapid characterization and classification. Here, we introduce ReporType, a versatile bioinformatics pipeline designed for targeted loci screening and typing of infectious agents. Developed using the snakemake workflow manager, ReporType integrates multiple software for read quality control and de novo assembly, and then applies ABRicate for locus screening, culminating in the production of easily interpretable reports for the identification of pathogen genotypes and/or screening of specific genomic loci. The pipeline accommodates a range of input formats, from Illumina or Oxford Nanopore Technology (ONT) reads (FASTQ) to Sanger sequencing files (AB1), or FASTA files, making it flexible for application in multiple pathogens and with different purposes. ReporType is released with pre-prepared databases for some viruses and bacteria, yet it remains easily configurable to handle custom databases. ReporType performance and functionality were validated through proof-of-concept exercises, encompassing diverse pathogenic species, including viruses such as measles, Newcastle disease virus (NDV), Dengue virus (DENV), influenza, hepatitis C virus (HCV) and Human T-Cell Lymphotropic virus type 1 (HTLV-1), as well as bacteria like *Chlamydia trachomatis* and *Legionella pneumophila*. In summary, ReporType emerges as a simple, dynamic and pan-pathogen tool, poised to evolve in tandem with the ever-changing needs of the fields of pathogen genomics, infectious disease epidemiology, and one health bioinformatics. ReporType is freely available at GitHub.

## 1. Introduction

The early identification and characterization of pathogen genetic variants is crucial for understanding the genetic diversity responsible for differences in the transmissibility and/or pathogenicity of a given infectious agent. Genomic surveillance is also critical to the study pathogens’ ecology, evolution over time, as well as the epidemiology of numerous infectious diseases they can cause [1,2,3,4,5,6,7], contributing to enhance their clinical diagnosis, treatment, and prevention [1,2,3,4,5,6,7]. In this context, the automation of routine bioinformatics workflows for the analysis of sequence data holds significant relevance, as reflected by the great efforts of the scientific community to develop and share new tools for user-friendly, intuitive and rapid pathogen sequence data analysis, classification and exploration [8,9,10,11,12,13]. In addition, as pathogen whole-genome sequencing (WGS) is rapidly becoming the gold-standard typing method, various bioinformatics tools have emerged for in silico prediction/capture of pheno- and genotyping data traditionally acquired with the old typing methods [14], such as tools for single- or multi-locus typing [15,16], serotype prediction [17,18], or virulence and antimicrobial resistance screening [19,20,21]. This accelerates the technological transition to more advanced sequencing technologies while maintaining compatibility with historical typing data. All these developments are of utmost relevance and utility not only for laboratories that already handle numerous daily sequences for clinical, surveillance, or research purposes, but also for laboratories taking initial steps in the realm of public health bioinformatics. Indeed, the lack of workflow automation and human resources with advanced training in WGS and bioinformatics, together with difficulties in keeping backwards compatibility with historical pathogen typing data, remain as primary bottlenecks in implementation of routine WGS-based pathogen surveillance. Recognizing the ongoing need for flexible tools capable of detecting loci of interest and/or different genotypes of pathogenic agents causing common infectious diseases in humans or animals, we introduce ReporType, which is a simple, reproducible and customizable bioinformatic snakemake workflow that can be shaped to several sequencing technologies, applications and pathogens.

## 2. Implementation

### 2.1. ReporType Architecture and Workflow

**Figure 1:**
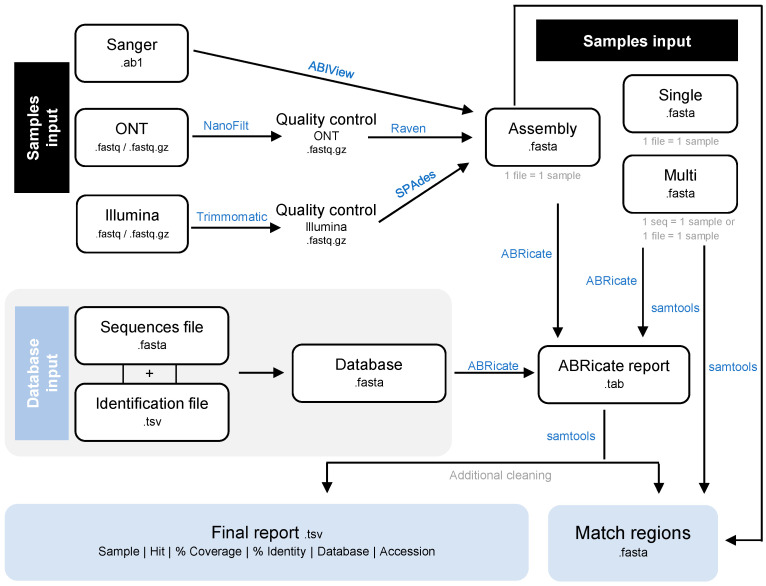
Schematic representation of ReporType data processing and analysis.

ReporType is a flexible tool designed for identifying loci of interest and/or determining genotypes of infectious agents, such as viruses and bacteria. This tool incorporates previously developed software for data processing and analysis (Figure 1 and Table 1), which are combined into a pipeline according to the analysis requirements using the Snakemake workflow manager, thus ensuring reproducible and scalable data analyses [22]. Moreover, it provides pre-prepared reference databases for locus screening/typing of some viruses and bacteria, being also easily configurable to handle custom databases (see below). As input files, ReporType accepts raw sequencing data, either from Sanger (AB1 format), Illumina or ONT technologies (FASTQ format, compressed or not), as well as single and multi-FASTA files, which are then processed and analyzed with a user-selected reference database. ReporType results are then presented in a main user-friendly tabular formatted file. Noteworthy, user-configurable parameters for each software enable a personalized analysis tailored to the species under investigation and the study goal.

ReporType pipeline begins by interpreting the user-provided inputs related to the reference database for locus screening and the samples for analysis, which are filtered and organized based on the respective sequencing technology and format. Samples can be supplied individually or placed in the same directory (even with different formats), thus ensuring optimal flexibility in input provision. As summarized in Table 1, raw Sanger sequences undergo filtering and processing using ABIView [23], read Illumina data undergo quality control via Trimmomatic [24], followed by de novo assembly with SPAdes [25], and read ONT data are quality filtered and trimmed by NanoFilt [26], with de novo assembly being conducted by Raven [27]. Following the pre-processing step, all FASTA files are analyzed with BLASTn through ABRicate [21], including those resulting from the de novo assembly or those directly provided as input, either as in single or multi-FASTA format. ReporType then performs additional filtering and cleaning of the ABRicate-generated reports in order to provide the essential information in a final user-friendly report (tsv format), including sample name, identified hit (loci/genotype), coverage and identity percentages for each hit, the analyzed database, and the unique identifier (accession) of the best match found in the database. ReporType also extracts (using SAMtools [28]) and saves the matching region in FASTA, indicating the start and end positions of the identified loci, which enhances the user’s understanding about the location of the targeted loci and facilitates downstream analysis (e.g., multi-sequence alignment and phylogenies). Intermediate output files, including original ABRicate reports, are kept and can be used for an enhanced output analysis and interpretation.

### 2.2. ReporType Installation, Configuration and Execution

ReporType is designed for installation and execution on UNIX systems or UNIX environments embedded in other operating systems, such as the Windows Subsystem for Linux (WSL) for Windows. The pipeline is also compatible with clusters, provided they support a Linux environment. ReporType can be installed via docker or conda, requiring the system to support the installation and execution of Conda, Python, Snakemake, and all other workflow-associated dependencies mentioned above and detailed in the GitHub repository https://github.com/insapathogenomics/reportype (accessed on 27 February 2024) [29]. After cloning the GitHub repository containing all the necessary configuration files and code for the pipeline, all installation steps are executed in the transferred directory.

The highly customizable parameters of ReporType in the “config” file (config.yaml) (Table 2) allow an analysis tailored to the investigated species and research objectives. As such, users need to configure the input parameters for their analysis either by pre-filling the ‘config.yaml’ file or by defining parameters via the command line. The provided default values are illustrative examples and should be adapted to the existing databases and input directories and formats. Given the broad utility of ReporType for multiple pathogens and research purposes, we anticipate that its common usage will involve creating a different config file for each analysis, comprising not only optimal parameters for a given database, but also for the input format and pathogen under study, thus running workflows for specific, reproducible and robust locus screening or pathogen genotyping. Crucial configuration parameters include ‘sample_directory’ and ‘input_format’ for the input samples and the database definition parameters ‘database’, ‘fasta_db’, and ‘table_db’ (database configuration details are described in the next section). The ‘sample_directory’ parameter requires the user to input the full path of the folder containing the files to be processed. This folder may contain input sequence data from different technologies and pre-prepared FASTA files (single or multi-fasta) for detecting loci in the available databases. Of note, in the case of directly submitting multi-FASTA files for analysis, ReporType interprets that each file corresponds to a single sample (as applied for de novo assembled contigs). Still, the user can request that each individual sequence within particular (or all) multi-FASTA files are handled as an independent sample by simply indicating the name of the files in the parameter “multi_fasta” in the config file. Importantly, if the same hit is detected multiple times in a sample, the user can specify in the ‘prioritize’ parameter which “best” hit should be included in the main report, based on either greater coverage (default) or greater identity.

The command line for executing ReporType pipeline exhibits considerable variability in complexity based on the configuration options desired by each user. If the input parameters are all defined through the config file, the simplest way to execute the pipeline involves a command consisting only of the tool’s name, followed by the number of CPUs to be used during the analysis ($ ReporType --cores all). If the user intends to configure ReporType execution via the command line, it is necessary to add the ‘--config’ argument before initiating the definition of the required input parameters. The simplest example of configuring the pipeline through the command line includes specifying the parameters to be changed in the configuration file (e.g., $ ReporType --cores all--config sample_directory=path/to/my_samples_folder/database=my_database). The GitHub repository [29] provides several usage examples, from the simplest to the most complex possible situations.

### 2.3. Database Configuration

ReporType is released with pre-defined databases for viruses and bacteria, yet it remains easily configurable to handle other databases shaped to any species or research purpose. To utilize a database that either comes with the installation or was previously prepared by the user, it is sufficient to specify its name in the configuration file or through the “database” parameter in the command line. If the targeted database has not been previously used with ReporType, the user must provide the complete path to the FASTA file containing the desired database, which must be already formatted according to the minimal ABRicate standards (“sequence~~~id~~~accession”), with sequence names excluding the character “-” (see GitHub for details). The database code name will correspond to the name of the provided FASTA file. Alternatively, if the user lacks a pre-formatted FASTA file for ABRicate database creation, ReporType can build one. For this, the user needs to provide as input the name of the new database (“database=my_database”), a FASTA file with the sequences (“fasta_db=path/to/sequences.fasta”) and a table (tsv format) identifying each sequence (“table_db=path/to/table.tsv”), with the following columns: (i) “sequence”, corresponding to sequence name in the provided FASTA file; (ii) “id”, which corresponds to the identification to be reported (e.g., gene name, lineage, subspecies, or other harmonized nomenclatures of the genotypes to be identified); and (iii) a unique identifier (e.g., NCBI accession) of each reference sequence that compose the database. It is recommended to create the directories of new databases within the same folder as the other existing databases. Noteworthy, ReporType can also accommodate typing nomenclatures including different levels (e.g., type and subtype). For this, users should identify the nomenclature level in the name of the database sequences, separated by the character “_” (e.g., “database_type~~~id~~~accession” for sequences defining the influenza A and B “types”, and “database_subtype~~~id~~~accession” for sequences defining the NA or HA subtypes). This flexible function has been tested and integrated for influenza type/subtype/lineage identification. Additional adjustments in the ‘table_configuration’ script may be required to adapt ReporType to more complex situations.

### 2.4. Databases, Test Datasets and Benchmarking

In order to test and demonstrate the applicability, flexibility and functionality of ReporType, we aimed at identifying case studies where ReporType could help reducing the need for laborious sequence handling (through “manual” alignment or BLAST) for pathogen genotyping or facilitating the transition from Sanger- to next-generation sequencing (NGS)-based genotyping. For this, we consulted several National Reference Laboratories (NRL) for pathogenic virus and bacteria of the National Institute of Health Dr. Ricardo Jorge (INSA) and the National Institute for Agricultural and Veterinary Research (INIAV) from Portugal, which besides advising on some case studies also indicated/provided useful databases and datasets. For some of the tested pathogens, databases and/or test sequences available in public repositories (ENA and NCBI), or previously analyzed in the literature, were also gathered to enrich ReporType benchmarking, as described below. In summary, for each proof-of-concept exercise (detailed below), the following data were collected: (i) a “database”, i.e., loci sequences representative of different genotypes (or serogroups, type, subtype, depending on the species) (detailed in Tables S1 and S2); and (ii) “test datasets” including sequences with known “genotype” classification to be used as control (detailed in Tables S3 and S4).

#### 2.4.1. Virus

Measles virus, which is a highly contagious virus that can cause a serious human airborne disease, is traditionally genotyped based on the genetic variability of a highly polymorphic region in the genome that is located in the gene encoding the nucleoprotein (N) [30]. Measles genotypes are designated by sequential numbers and letters, such as A, B1, D6, H2, E, F, among others [31]. In this study, an ABRicate sequence database was constructed based on the “Manual for the Laboratory-based Surveillance of Measles, Rubella, and Congenital Rubella Syndrome” [32], including representative sequences (n = 28) of the N gene from 24 genotypes. As a test dataset, we used: (i) all complete viral genome sequences (FASTA; n = 494) available at NCBI database with genotype information (https://www.ncbi.nlm.nih.gov/labs/virus/vssi/#/; taxid: 11234; consulted on 13 June 2023) [33]; (ii) ONT raw reads (SRR19430005-SRR19430012) of eight measles-positive samples from Uganda subjected to WGS through amplicon-based MinION sequencing, and classified as genotype B3 [34].

Newcastle disease virus (NDV) is a highly contagious virus, particularly devastating in immunologically naïve poultry [35], is traditionally genotyped based on the genetic variability of the fusion protein (F) gene [36]. In this study, an ABRicate sequence database for NDV was prepared based on the unified classification system and nomenclature described by Dimitrov and colleagues in 2019 [36], including F sequences (n = 1959) representative of all described sub/genotypes. As test datasets, we used: (i) F gene sequences (FASTA; n = 232) with genotype information described by Sun and colleagues [37], downloaded from the NCBI database; and (ii) partial F curated sequences (FASTA; n = 23) and raw Sanger sequences (AB1 format; n = 42) with known viral genotypes from the NDV sequence collection of the Virology Laboratory of INIAV, Portugal.

Dengue virus (DENV) can be transmitted to humans through the bite of infected mosquitos, occasionally causing severe disease, and even death. Its genome, comprising ~11,000 nucleotides, is translated into a single polyprotein that, upon cleavage, yields essential proteins for the production of new viral particles. DENV is traditionally classified into four “serotypes” (1, 2, 3 and 4), further divided into different genotypes [38] based on the variability of the polyprotein coding sequence. In this study, an ABRicate sequence database was prepared based on 145 representative serotype/genotype sequences comprehensively compiled by Mendes and colleagues [39], from which we also consulted 3830 complete DENV genomes (FASTA; n = 3830) and raw Illumina reads (FASTQ; n = 21) with known genotypes to be used as test datasets.

Influenza viruses are major human and/or animal pathogens that cause both seasonal endemic infections and periodic unpredictable pandemics. Four influenza types are defined (A, B, C and D), with the prevalent influenza A viruses being classified in 18 hemagglutinin (HA) subtypes and 11 neuraminidase (NA) subtypes, and influenza B being classified in two lineages, Yamagata and Victoria. ReporType was tested with an ABRicate database for rapid influenza type and subtype/lineage identification previously implemented into the web-based bioinformatics platform INSaFLU [11]. To prepare a test dataset, we also took advantage of the sequence dataset (~180,000 MP/M1 and HA/NA publicly available sequences for type and subtype/lineage identification, respectively) used as proof-of principle to implement the same ABRicate database in INSaFLU [11]. In addition, we also tested publicly available raw Illumina reads (FASTQ; n = 17) from seasonal A/H1N1 and A/H3N2 [40] and raw ONT reads (FASTQ; n = 14) corresponding to multiple influenza A subtypes [41].

Hepatitis C virus (HCV) can cause both acute and chronic hepatitis, ranging in severity from a mild illness to a serious, lifelong illness, including liver cirrhosis and cancer [42,43]. Genotypes are traditionally defined based on the genetic variability of core, E1, and NS5B regions [42,43], although other polymorphic regions, such as the NS4B and NS5A genes (targets of anti-viral resistance mutations), have also proven suitable for this purpose [43]. In the present study, we constructed two ABRIcate databases, one of them (“HCV_complete”) containing sequences from a fragment of the NS4B-NS5A region (n = 19), and the other (“HCV_partial”) containing only partial NS5A sequences (n = 19). This proof-of concept exercise strictly focused on testing samples analyzed in a recent article [43] conducted by the Portuguese NRL of HIV and hepatitis B and C, so it did not intend to target the whole-HCV genotype diversity. The test datasets included 83 samples with known HCV genotype, for which both genomic regions (NS4B-NS5A and partial NS5A) were provided by the NRL in raw Sanger format and respective FASTA (after manual curation) [43]. In addition, the consensus sequences generated from amplicon-based Illumina sequencing of the same samples [43] were also screened against both “HCV_complete” and “HCV_partial” databases. Of note, as the HCV databases do not cover the virus genotype diversity, they are available in an independent database folder (“databases_only_test”) in the GitHub repository.

Human T-cell lymphotropic virus type 1 (HTLV-1) is transmitted primarily through infected body fluids and can cause a type of cancer named adult T-cell leukemia/lymphoma (ATL). This virus is traditionally classified into three subtypes, A, B, and C, further subdivided according to the global regions where they prevail [44]. Each subtype and its respective subdivisions are traditionally defined based on differences on polymorphic loci, such as env and 5′ long-terminal repeat (LTR). Here, ReporType was tested using two sequence databases provided by the Portuguese NRL, one with *env* (n = 32) and the other LTR sequences (n = 43). The test datasets included 14 samples with known HTLV-1 subtype classification [45,46], for which both genomic regions (LTR and *env*) were provided by the NRL in raw Sanger format (AB1) and respective FASTA sequence after manual curation by the NRL [45,46]. Similar to the HCV database, as the HTLV-1 databases do not reflect the virus genotype diversity, they are available in an independent database folder (“databases_only_test”) in the GitHub repository.

#### 2.4.2. Bacteria

*Chlamydia trachomatis*, which is the causative agent of the most common sexually transmitted bacterial infection, is traditionally genotyped based on the variability of the gene *ompA*, which codes for its main antigen [47]. Fifteen major *ompA*-genotypes (A to L3) are currently defined. This traditional classification strongly correlates with tissue tropism and disease outcome: ocular disease (genotypes A, B/Ba, and C), anorectal and urogenital disease (D–K) and lymphogranuloma venereum (LGV) (genotypes L1–L3) [47,48,49]. In this study, we incorporated into ReporType the sequence database enrolling reference and variant sequences of the main *ompA* genotypes that is routinely used for *C. trachomatis* typing by the Portuguese NRL for Sexually Transmitted Infections at INSA [47,48]. As test datasets, we used: (i) partial ompA sequences with known genotype (as determined by the LNR), including raw Sanger sequences (AB1 format; n = 923) and FASTA sequences (after manual curation: n = 2208) from the INSA collection [47,48], and diverse complete *ompA* sequeces obtained through WGS [49]; (ii) publicly available WGS lllumina read data (FASTQ format; n = 524) covering a vast genome-scale diversity and all *ompA* genotypes [49,50,51,52]; and (iii) public WGS ONT data described in the literature (FASTQ format; n = 4) [50].

*Legionella pneumophila* is the causative agent of Legionnaires’ disease (LD), a severe pneumonia. WGS is now the preferred approach to support a more comprehensive detection and investigation of LD outbreaks and source attribution [53,54]. Consequently, efforts have been made to develop tools capable of extracting traditional typing data from WGS data, specifically the historically used sequence-based typing (SBT) profile [55,56]. In the present study, we investigated the ReporType application to infer *L. pneumophila* subspecies and serogroup from sequencing data. For this purpose, we constructed two ABRIcate databases: (i) “lp_serogroup_typing”, composed of *wzm* and *wzt* sequences to predict different *L. pneumophila* serogroups [57,58,59]; and (ii) “lp_subspecies_prediction”, including *gyrB* sequences (whose phylogenetic tree shows good correlation with the four currently defined subspecies) and sequences from five genes identified as unique for each subspecies (i.e., present in all *L. pneumophila* isolates of a select subspecies, but absent in all isolates of the other subspecies) by Kozak-Muiznieks and colleagues [60]. Therefore, ReporType in silico subspecies prediction relies not only on nucleotide identity in *gyrB*, but also on the presence/absence of other genes. As test datasets, we used: (i) draft and complete genome *L. pneumophila* sequences covering several serogroups or subspecies (FASTA; n = 26), which were run against the “lp_serogroup_typing” and “lp_subspecies_prediction” databses; and (ii) publicly available WGS lllumina read data (FASTQ format; n = 19) from strains with known *L. pneumophila* subspecies [60] to further test the “lp_subspecies_prediction” database. As an exploratory exercise, contrasting to the genotyping-oriented usages described above, we also sought to show ReporType’s applicability for screening the presence of genes of interest. For this, an additional ABRIcate database (“lp_dot_icm”) covering a vast repertoire of genes encoding the virulence-associated Dot/Icm type IVB secretion system (T4BSS) substrates was built (available at ReporType’s Github) and tested against the genome assembly of the PtVFX/2014 strain associated with a large LD outbreak in Portugal for which the Dot/Icm was previously characterized [59].

All ReporType databases described in this study are available in Github repository. We used the default ReporType config parameters in the proof-of-concept studies, with exception for those using the “lp_serogroup_typing” and “lp_subspecies_prediction” databases, for which we applied a “minid” of 70 and 90, respectively (recommended to avoid false positive hits in non-pneumophila species from the *Legionella* spp. genus). The pipeline execution was performed on an HP Laptop with an Intel(R) Core(TM) i7-1255U 12th generation processor, with 10 CPUs and 16 GB RAM. While the operating system used was Windows 11, the pipeline ran in a Linux Ubuntu environment created from the Windows Subsystem for Linux (WSL). The ReporType functionalities were further validated on the cluster available at the Institute of Biomedicine (IBiMed) at the University of Aveiro. This cluster comprises a server with 240 CPU cores, approximately 1.2 TB RAM and operates on the CentOS 7.7 OS managed by the Open Grid Engine with OpenMPI resources.

## 3. Results and Discussion

**Figure 2:**
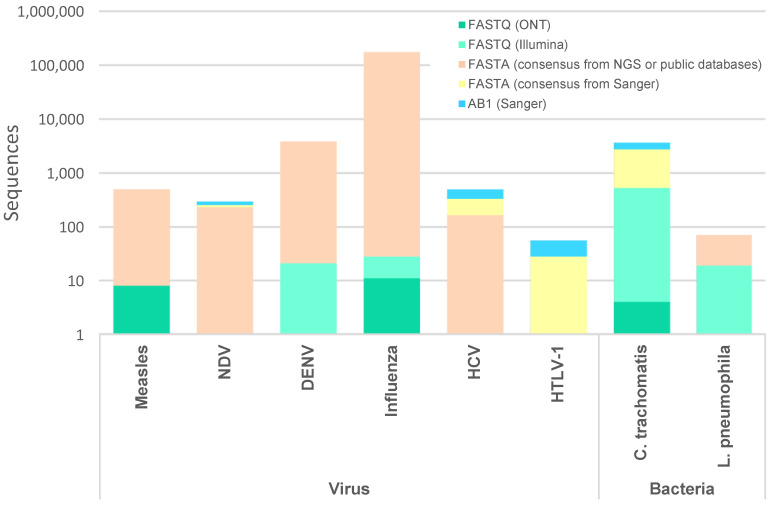
Summary of the number of sequences and input format covered in ReporType proof-of-concept exercises, per pathogen. Details are provided in Tables S3 and S4.

The development of automated, flexible and easily adaptable bioinformatics pipelines serves as a critical component in bridging the gap between cutting-edge sequencing technologies and practical applications in public health laboratories, enabling more efficient and informed responses to infectious diseases’ challenges. In order to address the need for simple and flexible tools for targeted loci screening and pathogen typing, we developed ReporType, a snakemake workflow from input sequence quality control and de novo assembly to ABRicate-based locus screening and reporting. Its performance, versatility and functionality was tested and validated through proof-of-concept exercises focused on showcasing applications where ReporType could streamline traditional Sanger sequence analysis, minimizing the manual effort of alignment or BLAST for targeted pathogen genotyping, or facilitate and promote the transition from Sanger to NGS-based genotyping by several NRLs in Portugal. These exercises covered vast sequence data from multiple viruses (measles, NDV, dengue, influenza, HCV and HTLV-1) and bacteria (*C. trachomatis* and *L. pneumophila*) and a high diversity of input formats (Figure 2), involving the construction of several reference databases (Tables S1 and S2) and analysis of several test datasets (Tables S3 and S4).

ReporType reached a 100% or nearly 100% success rate in reporting the expected classification (e.g., genotype, serogroup, type, subtype, depending on the screening goal and species) in all proof-of-concept exercises (Tables S3 and S4). The only two misclassifications were: (i) a very short Sanger AB1 sequence of HTLV-1 that only covered 56.4% of the *env* genotype representative sequences available in the reference “HTLV_1_env” database; (ii) the complete genome of *L. pneumophila* strain Thunder Bay (CP003730), for which ReporType reported serogroup (Sg) 12 (Tables S3 and S4) instead of the expected Sg6 [61]. Regarding the latter, a previous comparison between Sg6 strain Thunder Bay and Sg12 str. 570-CO-H optical maps determined that the O-antigen region is conserved between the two strains [61]. These data are aligned with our observation and suggests that, despite *wzt* and *wzm* are good genetic markers for discriminating *L. pneumophila* serogroups [57,58,59], specially the highly prevalent Sg1, certain atypical profiles (potentially generated by recombination) [62] may challenge an accurate in silico inference of specific serogroups, such as Sg6 and Sg12. In summary, ReporType proof-of-concept exercises clearly showed its good performance for several applications and pathogens, constituting an added-value not only to automate current genotyping workflows (either based on Sanger sequencing or NGS), but also to enhance laboratories’ flexibility to design and implement custom databases for specific loci screening or typing. On the other hand, these exercises consolidated and emphasized important and intuitive aspects to take into account when running ReporType or other BLAST-based tools. Specifically, results accuracy is, as expected, database-dependent, necessitating updates to pace with the known pathogen genetic diversity and dynamic typing nomenclatures. Indeed, it is important to keep in mind that the detection/typing is limited to “genotypes” present in the database, and, consequently, incomplete/out-of-date databases can cause misclassifications. This is valid not only for the tested and released databases, but also for the new databases that will be designed and incorporated at the user’s side. Fine-tuning critical parameters (e.g., minimum percentage of identity and coverage) for each situation (depending on the species, loci panel, type and goal of analysis, etc.) will also be necessary to ensure that the selected parameters are appropriately validated for up-to-date and accurate genotyping/screening. Discrepancies are expected to be more likely to rise in raw data analysis (Sanger, Illumina, or ONT), especially without stringent coverage and identity thresholds. For example, while raw Sanger sequence data were successfully tested here, the need and stringency of pre-curation steps will largely depend on the sequencing error rate and the reference database diversity, which again depends on the pathogen and the typing resolution that is needed. Regarding the de novo assembly step, optimization of specific parameters is also advisable to increase performance and efficiency according to the type of input data (NGS data from pure cultures, amplicon-based NGS, shotgun metagenomics, etc.). For instance, when the depth of coverage is too low or too high for Illumina (leading to high assembly fragmentation), or due to the still challenging performance of current assemblers for ONT data (such as, the implemented Raven), incomplete or unassigned classifications can be exacerbated. Due to its versatility (variety of input formats and workflows), ReporType runtime is expected to be strictly dependent on the type and size of input sequences (with NGS data being more time consuming than Sanger/FASTA), as well as to the individual performance of the incorporated software. In general, the ReporType’s execution times should be quite satisfactory and encouraging, showing that ReporType can be smoothly integrated into current genomic surveillance workflows without excessive computational time consumption. For instance, when samples in FASTA format are provided (expected common usage for ReporType), execution times are almost only dependent on ABRicate analysis, thus being remarkably faster. The reliance of ReporType on the snakemake workflow manager is expected to be an advantage for the future incorporation of alternative software for the existing analytical steps or new modules for new functionalities (alignment, phylogeny, etc.). Ultimately, user report exploration and interpretation remains crucial to ensure reliable coverage and identity percentages for accurate genotype classification or locus screening. In conclusion, ReporType is an automated, easy-to-use and flexible pipeline, for loci screening and typing. Its application can be particularly useful for rapid locus screening and/or genotyping of infectious agents, namely virus and bacteria.

## Figures and Tables

**Table 1:** ReporType input, software components and tasks.

Input Format (Sequencing Technology)	Software	Action(s)
AB1 (Sanger)	ABIView [23]	Trimming/Conversion to FASTA
FASTQ or FASTQ.gz (Illumina, single or paired-end)	Trimmomatic [24]	Quality control/Trimming
	SPAdes [25]	de novo assembly
FASTQ or FASTQ.gz (ONT)	NanoFilt [26]	Quality control/Trimming
	Raven [27]	de novo assembly
SINGLE or MULTI-FASTA (all)	ABRIcate [21]	Locus screening/typing and Reporting
ReporType tabular report (all)	SAMtools [28]	Extraction of match sequences

**Table 2:** ReporType main configuration parameters.

Configuration (Sequencing Technology)	Tool	Parameter
General (all)	ReporType	sample_directory
		input_format
		database
		(or ‘fasta_db’ and ‘table_db’ to setup a new database)
		output_name
		output_directory
		multi_fasta
		threads
		prioritize
General (all)	Snakemake	config
		np
		configfile
		snakefile
Specific (Sanger)	ABIView	startbase
		endbase
Specific (Illumina)	Trimmomatic	illuminaclip
		headcrop
		crop
		slidingwindow
		minlen
		leading
		trailing
		encoding
Specific (ONT)	Nanofilt	quality
		length
		maxlength
		headcrop
		Trailcrop
Specific (ONT)	Raven	Kmer
		polishing
Specific (all)	ABRicate	minid
		mincov

## Data Availability

ReporType source code is freely available via GitHub at: https://github.com/insapathogenomics/reportype (including usage examples) (accessed on 27 February 2024). Reference databases and test datasets are available in Supplementary Materials.

## Supplementary Materials

The supporting information can be downloaded at: https://www.mdpi.com/article/10.3390/ijms25063172/s1.
